# Effects of Exogenous Application of Indole-3-Butyric Acid on Maize Plants Cultivated in the Presence or Absence of Cadmium

**DOI:** 10.3390/plants10112503

**Published:** 2021-11-18

**Authors:** Kristína Šípošová, Eva Labancová, Danica Kučerová, Karin Kollárová, Zuzana Vivodová

**Affiliations:** 1 Institute of Botany, Plant Science and Biodiversity Centre, Slovak Academy of Sciences, Dúbravská cesta 9, 845 23 Bratislava, Slovakia; kristina.siposova@savba.sk; 2 Institute of Chemistry, Slovak Academy of Sciences, Dúbravská cesta 9, 845 38 Bratislava, Slovakia; eva.labancova@savba.sk (E.L.); danica.kucerova@savba.sk (D.K.); karin.kollarova@savba.sk (K.K.)

**Keywords:** antioxidant enzymes, auxin, growth, indole-3-butyric acid, maize, nutrients, reactive oxygen species

## Abstract

Auxins are plant hormones that affect plant growth, development, and improve a plant’s tolerance to stress. In this study, we found that the application of indole-3-butyric acid (IBA) had diverse effects on the growth of maize (*Zea mays* L.) roots treated without/with Cd. IBA caused changes in the growth and morphology of the roots under non-stress conditions; hence, we were able to select two concentrations of IBA (10^−11^ M as stimulatory and 10^−7^ M as inhibitory). IBA in stimulatory concentration did not affect the concentration of H_2_O_2_ or the activity of antioxidant enzymes while IBA in inhibitory concentration increased only the concentration of H_2_O_2_ (40.6%). The application of IBA also affected the concentrations of mineral nutrients. IBA in stimulatory concentration increased the concentration of N, K, Ca, S, and Zn (5.8–14.8%) and in inhibitory concentration decreased concentration of P, K, Ca, S, Fe, Mn, Zn, and Cu (5.5–36.6%). Moreover, IBA in the concentration 10^−9^ M had the most positive effects on the plants cultivated with Cd. It decreased the concentration of H_2_O_2_ (34.3%), the activity of antioxidant enzymes (23.7–36.4%), and increased the concentration of all followed elements, except Mg (5.5–34.1%), when compared to the Cd.

## 1. Introduction

Auxins are a group of plant hormones that affect and control many metabolic processes, including plant growth and responses to the environment [1]. Biosynthesis, polar transport, and the generation of auxin maxima play key roles in the coordination of the plant’s growth. The crosstalk between auxins and other substances, e.g., ethylene, cytokinins, gibberellin, is also an important part of the regulation of auxin production and transport in plants [2]. The crosstalk between auxins and reactive oxygen species (ROS) is integrated into a complex hormonal network that controls diverse aspects of plant growth and development [1].

ROS, such as superoxide radicals, hydrogen peroxide, singlet oxygen, and hydroxyl radicals, are present in every plant cell because they are continuously produced as unwanted by-products of various metabolic pathways, which are mainly localized in mitochondria, chloroplasts, and nitrogen-fixing nodules [3,4,5]. However, the concentrations of ROS are controlled, reduced, and scavenged by antioxidant enzymes [3,4]. Superoxide is converted to hydrogen peroxide (H_2_O_2_) and oxygen (O_2_) by superoxide dismutase (SOD, EC 1.15.1.1), and then H_2_O_2_ is converted to water (H_2_O) and O_2_ by ascorbate peroxidase (APX, EC 1.11.1.11), guaiacol peroxidase (EC 1.11.1.7), catalase (CAT, EC 1.11.1.6), and glutathione reductase (EC 1.8.1.7) [4,5]. All types of ROS are involved in the networks of signaling pathways and in the responses to environmental factors [3,4].

Cadmium (Cd) belongs to a group of environmental stress factors and is toxic not only for the plant but also for all living organisms [6,7,8]. The concentration of Cd in the environment in the last decade is increasing exponentially, mainly due to contaminated sewage sludge and wastewater leakage, or due to landfills [9]. Even the low concentration of Cd in the environment can be easily transferred from contaminated soils to plants, and enhanced accumulation of Cd^2+^ in the tissues poses a great risk to all living organisms through the food chain [10]. Once absorbed, Cd is retained in the human body and is toxic to the kidney, respiratory and digestive systems, and can cause bone demineralization and other diseases [11]. Exogenously applied auxins can alleviate Cd toxicity [12], but the mechanisms of their action are still not fully understood.

Bashri and Prasad [12] found that indole-3-acetic acid (IAA) increases the activity of the ascorbate-glutathione cycle. Similar results were also observed by Khan et al. [13] in their study, where the exogenous application of IAA stimulated the level of ascorbate and the activity of the ascorbate-glutathione cycle which enhanced the ability of tomato seedlings to counter the Cd-induced oxidative stress. Therefore, this may indicate the regulatory role of IAA in the ascorbate-glutathione cycle that may contribute to Cd alleviation. On the other hand, Demecsová et al. [14] found that IBA induces a high production of nitric oxide (NO) which effectively reduces high levels of superoxide via the formation of peroxynitrite (ONOO^−^). One of the pathways of NO production during IBA treatment is due to the conversion of IBA to IAA. For instance, Piacentini et al. [15] found that the application of IBA on Cd stressed rice effectively supplemented the NO levels in roots which had plummeted due to Cd toxicity. The hypothesis suggests that exogenously applied auxin is likely involved in several ways leading to the alleviation of toxicity.

As mentioned above, Cd decreases the uptake and accumulation of nutrients, which results in decreased plant biomass. One of the possible actions of auxins might be via an improved uptake of nutrients; however, little is known about the effects of exogenously applied auxins on the nutrient status of plants growing in both contaminated and non-contaminated conditions. Many studies focused on the effects of IAA—natural auxin—on the antioxidant defense system and mineral nutrients, with/without the presence of toxic metals [12,16,17]. However, IAA is not suitable as a component of fertilizers because it has low stability in solution and quickly degrades [18]. Furthermore, only one or a non-specified concentration of auxin was used in available studies [12,16,17].

The aims of this study were to investigate the effects of the exogenously applied auxin indole-3-butyric acid (IBA) on the morphology and physiology of maize (*Zea mays* L.) plants cultivated with or without Cd. Maize is a staple food that is grown and distributed worldwide. It is grown on different soils around the world including those that are contaminated with toxic elements. For that reason, it is necessary to study the methods that could improve the growth and quality of this crop. In our experiments, we determined the concentration of H_2_O_2_, the activity of antioxidant enzymes, and the changes in the uptake of mineral nutrients in these treatments: two IBA concentrations with different effects on the growth of the maize roots cultivated without Cd, Cd treatment, and one stimulatory concentration of IBA in the presence of Cd.

We chose IBA because it has higher stability in the solution than IAA [18]. IBA in the aqueous solution retains its stability for more than 28 days [19], while IAA disintegrates in the solution after 2 days. Stability of IBA depends on many factors such as oxygen level or exposure to the heat and light. IBA in the combination with alkylated choline cations is much more stable and may be suitable as fertilizer [20].

Plants were cultivated hydroponically during whole experiments. This type of cultivation allows a better examination of the root system but its disadvantages are abnormal root conditions [21]. Roots had not only different morphological properties (length, root hairs) but also anatomical (development of apoplasmic barriers), compared to the roots grown in the soil [22].

## 2. Results

### 2.1. Effects of IBA on the Growth Parameters in the Absence and Presence of Cd

**Figure 1:**
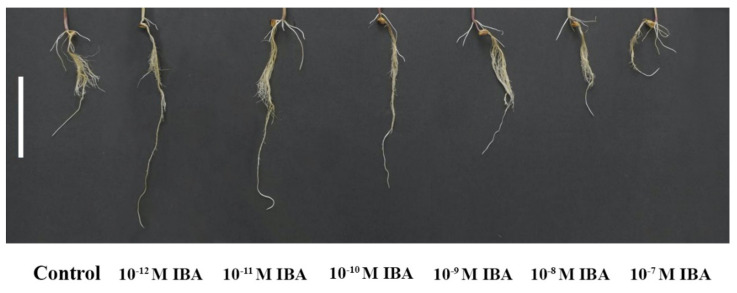
The effects of IBA on the root morphology of plants. Scale bar is 10 cm.

We tested the effects of IBA in different concentrations (range from 10^−12^ M to 10^−7^ M) and determined their effects on the growth parameters (Figure 1).

**Figure 2:**
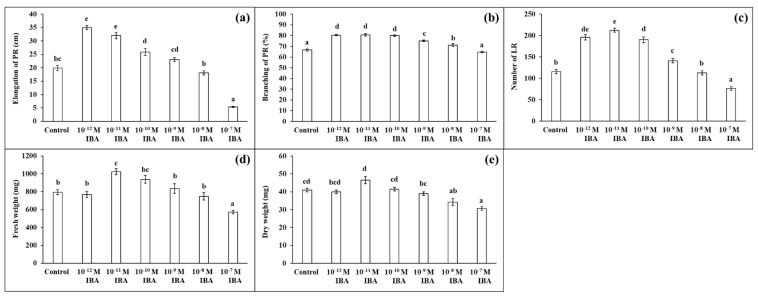
Elongation of PR (**a**), branching of PR (**b**), number of LR (**c**), fresh weight (**d**), and dry weight (**e**) of maize roots per one plant cultivated in Hoagland solution (control) and in the same medium supplemented with IBA in various concentrations (10^−12^–10^−7^ M). The data are presented as means ± standard error (*n* = 45). PR—primary roots; LR—lateral roots. Different letters denote statistically significant differences in the parameters between the treatments at *p* < 0.05 according to Tukey test.

IBA in concentrations of 10^−12^ M, 10^−11^ M, 10^−10^ M stimulated root growth compared to the control (Figure 2). The elongation of PR increased with decreasing auxin concentration (10^−10^ M—27.0%; 10^−11^ M—60.7%; 10^−12^ M—75.6%) (Figure 2a), and the strongest increase in PR branching (the length of the branched part of the PR) was found in those plants treated with 10^−12^ M, 10^−11^ M, 10^−10^ M (by 13.5%, 13.8%, 12.6%, respectively) (Figure 2b). The concentration 10^−9^ M had only a small stimulatory effect on root branching (by 8.2%). IBA also affected the number of LR. The highest number of LR was determined in the plants treated with IBA in concentrations of 10^−11^ M (by 83.2%), 10^−12^ M (by 68.8%), and 10^−10^ M (by 64.0%), compared to the control (Figure 2c). Similar to the root branching, IBA in a concentration of 10^−9^ M stimulated the formation of LR only slightly (by 21.9%). Even though, the application of IBA in stimulatory concentrations (10^−12^, 10^−11^, 10^−10^, 10^−9^ M) positively influenced the growth of the maize roots, only the 10^−11^ M concentration increased the FW of the roots significantly (by 28.8%) and none of the concentrations affected their DW to any great extent. IBA in the 10^−8^ M concentration had minimal effects on the growth parameters (Figure 2d,e). IBA in the 10^−7^ M concentration negatively affected plant growth: inhibited the elongation of PR (by 73.2%), decreased the number of LR (by 34.5%), FW, and DW (by about 27%) compared to the control. In the light of these results, we selected two concentrations of IBA that had different effects on root growth: 10^−11^ M as stimulatory and 10^−7^ M as inhibitory.

**Figure 3:**
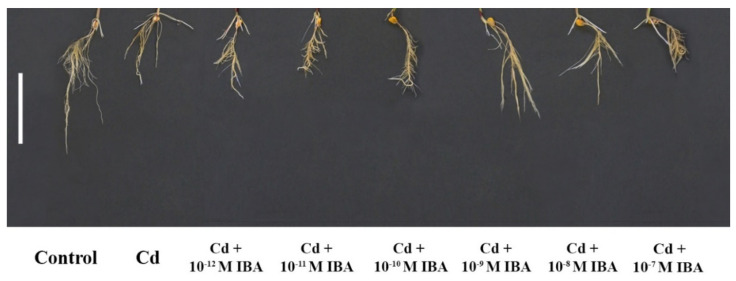
The effects of IBA on the root morphology of plants treated with Cd. Scale bar is 10 cm.

**Figure 4:**
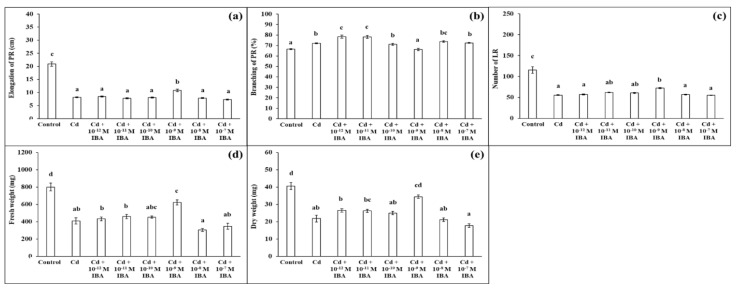
Elongation of PR (**a**), branching of PR (**b**), number of LR (**c**), fresh weight (**d**), and dry weight (**e**) of maize roots per one plant cultivated in Hoagland solution (control) and in the same medium supplemented with Cd (50 mM) or Cd and IBA in various concentrations (10^−12^–10^−7^ M). The data are presented as means ± standard error (*n* = 45). PR—primary roots; LR—lateral roots. Different letters denote statistically significant differences in the parameters between the treatments at *p* < 0.05 according to Tukey test.

We ascertained that Cd in the 50 μM concentration in the substrate negatively affected plant growth (Figure 3). Cd strongly inhibited elongation of PR (by 60.8%), decreased the number of LR (by 52.1%), FW (by 48.8%), and DW (by 46.3%), when compared to the control (Figure 4). However, Cd stimulated branching of the PR (by 8.1%).

We tested the effects of IBA in the same concentrations as above (ranging from 10^−12^ M to 10^−7^ M) on plants growing in the presence of Cd (Figure 4). Contrary to the non-stress conditions, IBA in the 10^−9^ M concentration had the most significant stimulatory effects on plants under Cd stress (Figure 4). IBA in this concentration stimulated the elongation of PR (by 31.8%) (Figure 4a), the number of LR (by 30.7%) (Figure 4c), and both FW (by 52.1%) and DW (by 57.8%) (Figure 4d,e), compared to the Cd treatment. Concentrations that were lower or higher than 10^−9^ M did not significantly positively affect plant growth and we concluded that these concentrations were not suitable to be efficient in the alleviation of Cd toxicity.

### 2.2. Effects of IBA and Cd on the Concentrations of H_2_O_2_ and on the Activity of Antioxidant Enzymes

In our following experiments, we determined the changes in the concentration of H_2_O_2_ and the activities of three antioxidant enzymes (SOD, CAT, APX) in the plants that were treated with two different concentrations of IBA (10^−11^ M—stimulatory and 10^−7^ M—inhibitory), Cd in a 50 µM concentration, and a combination of Cd and the most effective concentration of IBA (10^−9^ M) in alleviating the toxicity of Cd (Cd + 10^−9^ M IBA treatment).

**Figure 5:**
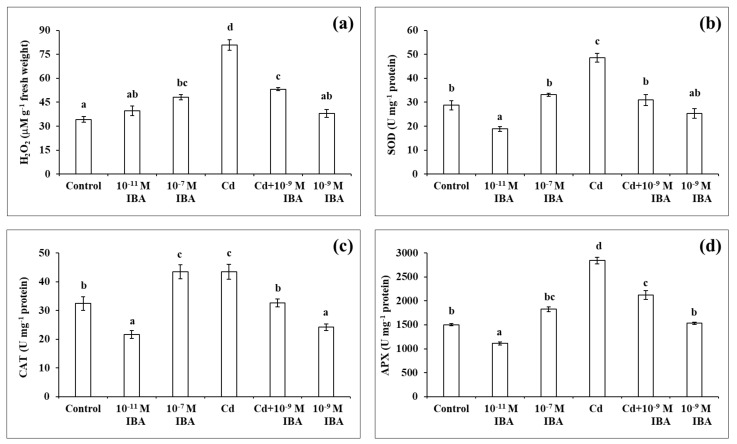
The concentration of H_2_O_2_ (**a**) and the activities of antioxidant enzymes SOD (**b**), CAT (**c**), APX (**d**) in the maize roots treated without/with Cd. The data are presented as means ± standard error (*n* = 15). Different letters denote statistically significant differences in the parameters between the treatments at *p* < 0.05 according to Tukey test.

The highest concentration of H_2_O_2_ was detected in the roots that had the most severe inhibition of root growth when compared to control (Figure 5a). The application of only Cd resulted in the highest increase in the concentration of H_2_O_2_ (by 136.5%), while the 10^−7^ M IBA treatment increased the concentration by 40.6%. Even though the concentration of H_2_O_2_ was reduced considerably in the Cd + 10^−9^ M IBA treatment (2.5 times lower than in the Cd treatment), it was still higher (by 55.5%) than in the control. We did not determine any significant differences between the control and the plants treated with other concentrations of IBA.

In plants treated only with auxin, the highest activity of all the enzymes studied was ascertained in the 10^−7^ M IBA treatment (Figure 5b–d). The activity of SOD, CAT, and APX increased by almost 15.3%, 34.1%, and 22.0%, respectively, when compared to control. The 10^−11^ M IBA treatment resulted in a decrease in the activity of SOD (by 34.2%). Our results indicate that the effects of IBA on enzyme activity depend on the concentration used.

In the plants treated with Cd, the highest enzyme activity was observed in the Cd treatment (Figure 5b–d). The activity of SOD, CAT, and APX increased by 69.2%, 34.2%, and 89.0%, respectively, when compared to the control. The Cd + 10^−9^ M IBA treatment decreased the activity of SOD (by 36.4%), CAT (by 25.1%), and APX (by 25.0%) when compared to the Cd treatment. The 10^−9^ M IBA treatment decreased only the activity of CAT (by 27.0%) compared to the control.

### 2.3. Effect of IBA on the Concentration of Cd

**Figure 6:**
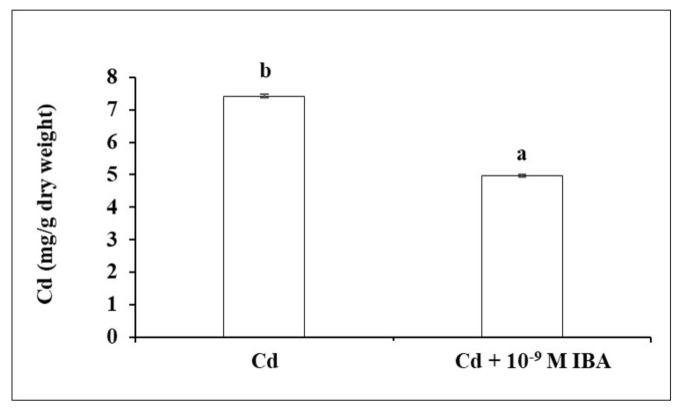
The concentration of Cd in maize roots of one plant treated without/with IBA. The data are presented as means ± standard error (*n* = 5). Different letters denote statistically significant differences in the parameters between the treatments at *p* < 0.05 according to Student’s *t*-test.

In our study, exogenously applied auxin decreased the concentration of Cd by 33.0% in the roots (Figure 6) when compared to the Cd treatment.

### 2.4. Effects of IBA and Cd on the Concentration of Mineral Nutrients

**Figure 7:**
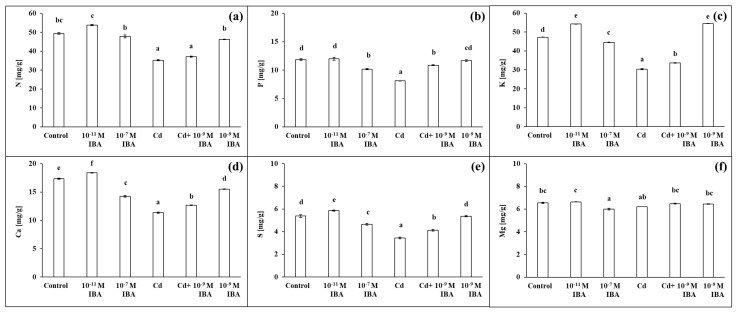
The concentrations of N (**a**), P (**b**), K (**c**), Ca (**d**), S (**e**), Mg (**f**) in maize roots treated without/with Cd. The data are presented as means ± standard error (*n* = 9). Different letters denote statistically significant differences in the parameters between the treatments at *p* < 0.05 according to Tukey test.

**Figure 8:**
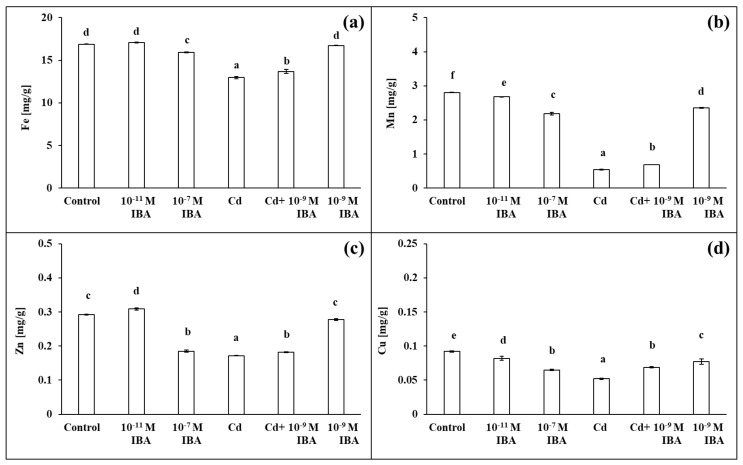
The concentrations of Fe (**a**), Mn (**b**), Zn (**c**), Cu (**d**) in maize roots treated without/with Cd. The data are presented as means ± standard error (*n* = 9). Different letters denote statistically significant differences in the parameters between the treatments at *p* < 0.05 according to Tukey test.

The two IBA concentrations (10^−11^ and 10^−7^ M) caused various changes in the concentrations of macro- and micronutrients in roots (Figure 7 and Figure 8). The 10^−11^ M IBA treatment increased the concentrations of N (by 8.9%), K (by 14.8%), Ca (by 6.1%), S (by 9.0%), and Zn (by 5.8%) and decreased the concentrations of Mn (by 4.5%) and Cu (by 10.9%) when compared to control. The 10^−7^ M IBA treatment decreased the concentrations of all nutrients, except for N. The concentrations of macronutrients in the 10^−7^ M IBA treatment decreased in the range between 5.7 and 17.9%, and the concentrations of micronutrients in the range between 5.5 and 36.6%.

The concentrations of all the selected macro- and micronutrients in the maize roots decreased in the Cd treatment, except Mg (Figure 7 and Figure 8). The concentration of Mn was the most affected, as it was decreased by 80.7% when compared to control (Figure 8b). Other nutrients that were greatly influenced were K, S, Cu, and Zn (Figure 7c,e and Figure 8c,d). The application of Cd decreased their concentrations in roots approximately by 40% when compared to control. The concentrations of N, P, and Ca decreased approximately by 30% (Figure 7a,b,d), and the concentration of Fe by 23.1% (Figure 8a). On the other hand, in the combined Cd + 10^−9^ M IBA treatment the concentrations of all above-mentioned nutrients increased in comparison to Cd treatment, except for Mg (Figure 7b–e and Figure 8). The exogenously applied 10^−9^ M IBA in the Cd + 10^−9^ M IBA greatly influenced the concentrations of P (by 34.1%), Cu (by 32.7%), S (by 20.0%), and Mn (by 26.8%) (Figure 7b,e and Figure 8b,d). Other nutrients were increased slightly (N—5.5%; K—10.8%; Ca—11.5%; Fe—5.5%; Zn—5.8%) (Figure 7a,c,d and Figure 8a,c). The 10^−9^ M IBA treatment increased the concentration of K (by 13.2%) (Figure 7c) and decreased the concentration of Ca (by 10.5%), Mn (by 16.0%), and Cu (by 16.8%) compared to the control (Figure 7d and Figure 8b,d).

### 2.5. Data Visualization and Analysis—Relationship among Different Treatments and Observed Parameters

**Figure 9:**
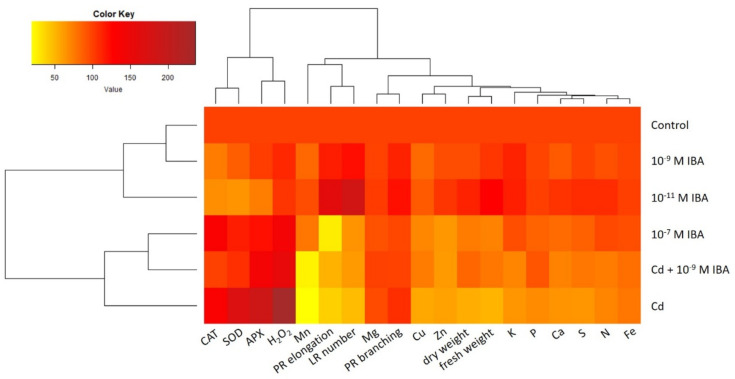
The heatmap analysis of the parameters studied in the treatments: control, 10^−11^ M IBA, 10^−7^ M IBA, Cd, Cd + 10^−9^ M IBA, 10^−9^ M IBA. The same color indicates a similar value of the parameter. Every value of every parameter is expressed as a percentage of its corresponding control. PR elongation-elongation of primary root; PR branching-branching of primary root; LR number-number of lateral roots; fresh weight-root fresh weight; dry weight-root dry weight.

A heatmap with dendrograms was used to analyze and visualize the data from the study (Figure 9). The dendrograms sort the treatments used in our study (control, 10^−11^ M IBA, 10^−7^ M IBA, Cd, Cd + 10^−9^ M IBA, 10^−9^ M IBA), as well as the parameters studied (growth parameters, activity of antioxidant enzymes, concentration of H_2_O_2_ and of nutrients) according to their similarity.

The lateral dendrogram reveals a similarity between the control and the 10^−9^ M IBA treatment. Both the control and the 10^−9^ M IBA treatment are grouped in a cluster together with the 10^−11^ M IBA treatment. The heatmap shows that these treatments had mostly positive or no effects on the parameters studied. Another cluster is formed by the treatments: Cd, Cd + 10^−9^ M IBA, and 10^−7^ M IBA, which share common features. The coloring clearly shows the increase in the activity of antioxidant enzymes, decrease in the growth parameters, and concentrations of nutrients in comparison with the control. It is noteworthy that there is a closer relationship between 10^−7^ M IBA and Cd + 10^−9^ M IBA treatments than between the Cd + 10^−9^ M IBA and Cd treatments.

**Figure 10:**
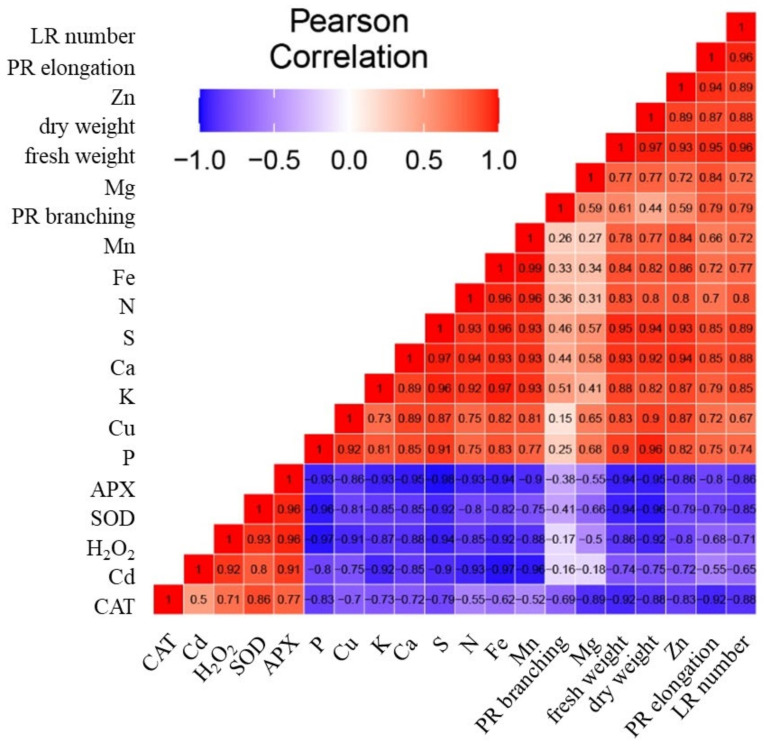
The correlation matrix heatmap shows the values of the Pearson correlation coefficient for all studied parameters, the positive values are in red, negative are in blue. The coefficient ranges from −1 (perfect negative linear relationship) to 1 (perfect positive linear relationship), while the value 0 indicates that there is no relationship between studied variables. The parameters analyzed were the elongation of primary root (PR elongation), PR branching, number of lateral roots (LR number), fresh and dry weight, the activity of enzymes APX, CAT, SOD, the concentration of H_2_O_2_, the concentrations of nutrients Ca, K, Mg, P, N, S, Fe, Mn, Cu, Zn, and the concentration of Cd.

Whereas the upper dendrogram of the heatmap indicates the relationships among parameters (Figure 9), the correlation matrix heatmap offers an insight into the correlation among them (Figure 10). The strong positive correlation was detected between the activity of antioxidant enzymes and the concentration of H_2_O_2_ (Figure 10). On the other hand, these parameters correlate negatively with the growth parameters and the concentration of nutrients. A strong correlation was detected among the concentrations of nutrients (with the exception of Mg) and biomass (fresh weight and dry weight). The highest Pearson coefficient values (range from 0.8 to 0.96) was detected between the biomass (fresh weight and dry weight) and the concentrations of P, S, Ca, Cu, K, Fe, and N.

The concentration of Cd correlates positively with the activity of antioxidant enzymes and the concentration H_2_O_2_. However, the concentration of Cd had strong negative correlation with nutrients, with the lowest Pearson coefficient values (range from −0.8 to −0.97) between the concentration of Cd and the concentrations of Fe, Mn, N, K, S, and P.

## 3. Discussion

The exogenously applied auxins affect plant growth via endogenous IAA levels by modifying the metabolism of IAA and changing their conjugations with other molecules [23]. The effects of auxins are highly dependent on their concentration. Very low concentrations of exogenous auxins do not cause these modifications. On the other hand, high concentrations of exogenous auxins increased the internal levels of IAA [18], which in turn induced the production of ethylene [24]. High concentrations of ethylene stop plant cell division and slow cell expansion [24] by alkalization of the apoplast, which subsequently decreases the activity of the enzymes responsible for extensibility of the cell wall [25]. Similarly, we observed that a high concentration of IBA (10^−7^ M) inhibited maize growth while a low concentration (10^−11^ M) had a stimulating effect. Marquez et al. [26] also observed that the habitus of maize roots depend on the type of auxin (IAA, IBA, NAA) and its concentration. Furthermore, new study proved that exogenously applied auxins induce the transverse microtubule organization in plant cells. This step is controlled during the interaction between auxin receptors and the nucleus [27]. The role of microtubules in the cellulose microfibril organization in plant cell walls is also known—transverse microtubules guide the cellulose synthase complexes [28]. It was ascertained that exogenously applied auxin also causes the changes of the cell wall composition of maize roots [29]. Auxin mainly increases the content of cellulose and Klason lignin, components responsible for the strength of the cell walls.

As mentioned above, exogenously applied auxins also alleviate the toxic effects of Cd [12] and improve the tolerance of plants to stress [30]. The presence of Cd in plant cells has damaging effects on biomolecules, which are important for many physiological processes [31]. Cd in short term, as well as in long term treatments [30,32] decreases the internal level of IAA because it induces IAA degradation by stimulating the activity of IAA-oxidase [12]. Cd also alters the expression of several important auxin biosynthetic and catabolic genes [6]. Thus, the negative effects of Cd on root growth determined in our experiment might also be connected to these processes. In our study, exogenously applied IBA might have increased the internal level of auxin (previously reduced under the Cd treatment), which resulted in stimulation of the plant growth. The need to increase the internal level of auxin concentrations in the roots might explain the differences between the most efficient concentration of IBA under non-stress conditions (10^−11^ M) and the most efficient concentration of IBA under Cd stress (10^−9^ M).

Another toxic effect of Cd is an alteration of oxidant level of plants through the overproduction of ROS, such as H_2_O_2_ [33,34]. The molecules of H_2_O_2_ originate from the process of cell respiration in the mitochondria [35], and the low concentrations of H_2_O_2_ in the root cells act as signaling molecules and regulators of the expression of some genes, and in many aspects resemble phytohormones. A rise in the concentration of H_2_O_2_ damages cell membranes, as well as the processes of respiration, and indicates oxidative stress in plants [12,35]. Oxidative stress occurs in plants exposed to suboptimal growth conditions, e.g., the presence of pollutants.

Auxins have a close association with ROS because these molecules are able to interact with each other [36]. This crosstalk is easily disturbed by changes in the concentrations of auxins or ROS and can result in an impairment of plant growth and development. In our study, the highest concentration of exogenously applied auxin inhibited root growth and increased not only the concentration of H_2_O_2_ but also the activities of APX and CAT. Similarly, Bashri and Prasad [12] observed a higher concentration of H_2_O_2_ and elevated antioxidant enzyme activity after treatment of fenugreek (*Trigonella foenum-graecum* L.) seedlings with IAA in a concentration that inhibited their growth. Externally applied auxin probably increases the internal level of auxin and subsequently changes the cellular oxidative status of the plant [36], resulting in an increase in ROS production [12]. Hence, the balance between produced-ROS and scavenged-ROS is disturbed and the root cells activate antioxidant enzymes [30]. In our experiment, the stimulatory concentration of IBA (10^−11^ M) did not affect the concentration of H_2_O_2_ and decreased the activity of all the enzymes studied. El-Gaied et al. [37] also observed a decrease in *SOD, CAT,* and *TPX* gene expression after treatment with IAA and IBA in stimulatory concentrations. On the other hand, Bashri and Prasad [12] observed an increase in the activities of SOD, APX, and CAT despite a reduction in the concentration of H_2_O_2_. The effects of auxin on the activity of antioxidant enzymes depended not only on the concentrations used, but also on the type of the auxin used [37], and the age of the plants [17]. Various activities were also found in different zones of the primary root [38]. The plant species, its phenotype, and its defense mechanisms possibly play an important role in the reaction of antioxidant enzymes to the exogenously applied auxin.

Heavy metals might elevate the levels of H_2_O_2_ in the cells which are linked with the enhanced transport of IAA through auxin transporters as a response to damaged plant cell metabolic pathways. Thus, the H_2_O_2_ levels are directly connected to the stress state of the plants [39]. In our experiments, the presence of Cd in the maize roots increased the concentration of H_2_O_2_ and subsequently the activity of antioxidant enzymes: SOD, CAT, and APX. The same results were ascertained in plants treated with toxic metals [40]. All three enzymes are the first line of defense against oxidative stress [41] because they are responsible for the maintenance of the steady-state level of superoxide radicals and H_2_O_2_ [41].

In our experiments, the presence of IBA in combination with Cd decreased the concentration of H_2_O_2_, the activity of SOD, CAT, and APX, when compared to the Cd treatment. The addition of IBA to the medium might have alleviated the toxicity of Cd in two possible ways. The first way is that IBA could have scavenged the Cd in the cytoplasm. It is known that exogenous auxin can act as a chelating agent [42] because auxin occurs in a deprotonated form in the cytoplasm, which has a high affinity to Cd. The second way is that exogenously applied auxin probably supplements the reduced internal levels of auxin, which can affect the level of signaling molecules, for example NO, [14,15] and can be also involved in the signaling pathways of the defense mechanisms [30], for example biosynthesis of metal-binding ligands, phytochelatins, and glutathione [42]. The theory about the auxin signaling pathway in the defense mechanisms is also supported by our results. Exogenously applied IBA decreased the accumulation of Cd in the roots. Similar effects of stimulatory concentration of IBA on the Cd accumulation in chickweed (*Stellaria media* L.) and black nightshade (*Solanum nigrum* L.) roots was observed by Lin et al. [43] and Ran et al. [44]. Natural auxin, IAA, in plants of wheat (*Triticum aestivum* L.) [45], tamarillo (*Cyphomandra betacea* Cav.) [46], and tomato (*Lycopersicum esculentum* Mill.) [13] also decreased Cd concentration. On the other hand, the synthetic auxin (NAA) increased the concentration of Cd in arabidopsis (*Arabidopsis thaliana* L.) [32]. However, the plants grown in hydroponics grow differently from the ones that grow in soil. The delayed maturation of endodermis changed availability of nutrients as well as toxic metals, resulting in a different ion uptake. Still, the usage of hydroponics in basic research ensures constant conditions for plants [22].

Auxin can significantly affect the uptake and the concentrations of some nutrients by affecting proton pump ATPase [47]. In our study, the IBA in the stimulatory concentration (10^−11^ M) induced the uptake and accumulation of mineral nutrients (N, K, Ca, S, Zn) as opposed to the IBA in the inhibitory (10^−7^ M) concentration, which decreased their concentrations in roots (except for N). The changes in the uptake and accumulation of mineral nutrients in the roots after IAA treatment were also ascertained in pepper (*Capsicum annuum* L.)*,* alfalfa (*Medicago sativa* L.), and maize [16,17,47]. In all three plants, they observed significant changes, mainly in the concentrations of K, Ca, Mg, Mn, and Zn. They have not detected any changes in the concentrations of N, P, S, and Fe. Auxin probably influences mainly the nutrients that are involved with the growth of roots or the biosynthesis of auxin. The change in the uptake of these nutrients caused by auxins might depend on the plant species and/or duration of auxin exposure. The effects of IBA on the accumulation of mineral nutrients in the roots have not yet been studied.

The present study confirms that Cd affects the uptake of macro- and micronutrients. We ascertained that Cd treatment caused an exceptionally high decrease in the concentration of Mn compared to the control. Furthermore, the concentrations of the nutrients Cu and Zn were also highly reduced. The reductions in the concentrations of Mg and Fe were lower. Cd enters the root cell through essential nutrient transporters (for more information, see [48]). Higher concentrations of Cd in the soil increase the competition with essential nutrients for transporters present at the root surface, thus decreasing their uptake. Our results also show that the Cd + IBA treatment increased the uptake and accumulation of all nutrients, except for N. Changes in the uptake of mineral nutrients in the stressed plants of alfalfa and maize after IAA treatments, were also studied by López et al. [16] and Wang et al. [17]. López et al. [16] did not determine any significant changes caused by the IAA treatment in the plants stressed by Pb. Wang et al. [17] found that exogenously applied auxin decreased the concentrations of K and Mn after long treatment with auxin. The effects of auxin depend not only on the concentration used and the period of the exposure [17] but also on the concentration of the toxic metal [16]. In both mentioned studies, the effects of IAA (in the used concentration) were not linked with growth parameters as opposed to our study, in which the effects of both stimulatory and inhibitory concentrations of IBA are supported by the results of maize growth (Figure 2 and Figure 3).

The heatmap (Figure 9) shows the differences between the effects of Cd, Cd + 10^−9^ M IBA, 10^−11^ M and 10^−7^ M IBA treatments. Whereas negative effects of Cd on growth parameters are associated with the strong increase in the concentration of H_2_O_2_ and activity antioxidant enzymes and with the decrease in the concentrations of micro- and macronutrients; negative effects of 10^−7^ M IBA treatment do not include a significant decrease in concentrations of mineral nutrients. The positive effects of Cd + 10^−9^ M IBA treatment can be linked with increased concentrations of mineral nutrients and decreased activity of antioxidant enzymes. Moreover, the positive effect of 10^−11^ M IBA treatment on the growth parameters and concentrations of nutrients in comparison with the control is clearly visible.

The identification of the most efficient concentration of exogenously applied IBA is a very important result that might be applied in future methods used in the stimulation of plant growth. The usage of natural substances that are termed as biostimulants is quickly becoming a new trend in agriculture. The knowledge that an appropriate concentration of IBA can alleviate the negative effects of toxic elements that are present in the environment, including soils, is crucial for biotechnology. Furthermore, the stimulatory concentration of IBA used both in contaminated and non-contaminated conditions could improve the nutritional value of the plants. However, some portion of the heavy metals that had been uptaken by plants into their root system might leak into the soil and cause secondary pollution [49]. Therefore, basic methods of plant biomass recycling are not applicable in this instance. Some new techniques of dealing with the plant waste residues after phytoremediation seem promising and efficient [50]. For example, Danelli et al. [51] found that anaerobic digestion or combustion (e.g., pyrolysis and gasification) of polluted biomass could be used to produce energy and simultaneously as a remedy for the areas contaminated by Cu, Zn, and Cd. The production of energy from such material is considered as an environmentally friendly and sustainable process [52]. Furthermore, the application of auxins or other plant growth regulators is also starting to be a widely accepted and acknowledged approach for improving the methods of phytoremediation efficiency in contaminated areas.

## 4. Materials and Methods

### 4.1. Plant Material and Cultivation

Maize grains (hybrid Almansa) were obtained from RWA Slovakia s. r. o. Bratislava, Slovakia. The permits have been obtained for working with plants. We prepared and cultivated the plant material according to our previous research [29]. The maize grains were surface sterilized and imbibed for 3 h in distilled water in the dark at 25 °C. Then grains germinated for 3 days in wet perlite in the dark at 25 °C. After 3 days, seedlings were transferred to dark containers (3 l/15 plants) which contained Hoagland solution [53], or Hoagland solution supplemented with IBA in concentrations ranging from 10^−12^ M to 10^−7^ M, and with/without Cd(NO_3_)_2_ at a concentration of 50 μM, at pH 6.2, under controlled conditions (photosynthetic photon flux of 130–140 µmol m^−2^ s^−1^, 25/20 °C temperature, 16 h photoperiod, and 70% humidity) for 10 days. Continuous aeration of solution was given through an air pump by making bubbles. Hoagland solution was changed on the 5th day of cultivation.

### 4.2. Measurement of the Growth Parameters

After 10 days of cultivation, the healthy and undamaged plants were harvested. The elongation of the primary roots (PR) (the difference between the final length and the initial length of PR), the branching of the PR (the length of the branched part of the PR), the number of lateral roots (LR), and the fresh (FW) and dry weight (DW) of roots were determined. All growth parameters were calculated per one root. The roots were frozen individually in liquid nitrogen and stored at −70 °C until enzyme extraction. The roots used for elemental analyses were dried individually for 72 h at 105 °C. For our next analyses, we chose root or mix of roots of one treatment based on their growth parameters (the shortest and longest roots from every treatment were excluded from the mix).

### 4.3. Determination of H_2_O_2_

The hydrogen peroxide (H_2_O_2_) concentration was determined according to the modified method of Velikova et al. [54]. The maize roots (500 mg) were homogenized in a cold 50 mM sodium phosphate buffer (pH 7.0), then centrifuged at 5300 g for 10 min at 4 °C. The supernatant was diluted with 1 mM potassium iodide in the ratio 1:2. The absorbance was measured spectrophotometrically at 390 nm and the concentration of H_2_O_2_ was calculated based on a standard curve.

### 4.4. Determination of Antioxidant Enzymes Activity

The frozen roots (2.7 g fresh weight) were homogenized in liquid nitrogen and suspended in a 50 mM sodium phosphate buffer (7 mL, pH 7.8), containing 50 mM EDTA and protease inhibitor cocktail tablets (Roche Diagnostics GmbH, Germany). The homogenate was centrifuged at 3800 g for 30 min at 4 °C, and a supernatant was used to determine both the activity of the antioxidant enzymes and the concentration of soluble proteins. The latter was determined by the Bradford method, using bovine serum albumin as a standard [55].

The activity of SOD was determined according to Madamanchi et al. [56] and was measured spectrophotometrically at 560 nm. The reaction mixture contained a 50 mM sodium phosphate buffer (1.8 mL, pH 7.8), 0.15 mM MTT (150 µL), 13 mM methionine (600 µL), 1 mM EDTA (150 µL), and 2 µM riboflavin (150 µL). The mixture was placed in sample tubes under fluorescent light (50 µmol m^−2^ s^−1^) for 15 min. One unit of SOD activity is the amount of proteins causing a 50 % MTT reduction under the light and is expressed as U mg^−1^ protein.

The activity of APX was determined according to Nakano and Asada [57] and was measured spectrophotometrically at 290 nm. The reaction mixture contained a 50 mM sodium phosphate buffer (pH 7.0), 1 mM EDTA (300 µL), 0.5 mM ascorbate (300 µL), and 0.1 mM H_2_O_2_ (300 µL). One unit of APX is the amount of enzyme required to decompose 1 μM of ascorbate per 1 min and is expressed as U mg^−1^ protein.

The activity of CAT was determined according to Hodges et al. [58] and was measured spectrophotometrically at 240 nm. The reaction mixture contained a 50 mM sodium phosphate buffer (2 mL, pH 7.8) and 3% H_2_O_2_ (150 µL). Specific CAT activity was calculated according to Claiborne [59] and expressed as the amount of enzymes required to decompose 1 μM of H_2_O_2_ per 1 min and it was expressed as U mg^−1^ protein.

### 4.5. Determination of Selected Mineral Nutrients

Dried maize roots (250 mg) were dissolved in concentrated HNO_3_ (4 mL) and H_2_O_2_ (2 mL), and the solution was heated at 220 °C under pressure at 60 bar for 20 min. Measurements of the concentrations of macro- (Ca, K, Mg, P), micronutrients (Fe, Mn, Cu, and Zn), and Cd were carried out by flame atomic absorption spectrometry (AAS Perkin Elmer 1100 and 4100) and by inductively-coupled plasma mass spectrometry (ICP-MS, Thermo iCap Q) at the Institute of Laboratory Research on Geomaterials, Faculty of Natural Sciences, Comenius University in Bratislava, Slovakia. The concentrations of the macronutrients N and S were estimated by gas chromatography, using a FLASH 2000 Organic elemental analyzer (CHNS-O) from Thermo Fisher Scientific, Massachusetts, USA, at the Analytical Department, Institute of Chemistry SAS, Bratislava, Slovakia.

### 4.6. Data Visualization and Analysis—Heatmap and Correlation Matrix Heatmap

To create a heatmap and compare the parameters (elongation of PR, branching of PR, number of lateral roots, fresh and dry weight, the activity of enzymes: APX, CAT, SOD, the concentration of H_2_O_2_, the concentrations of nutrients Ca, K, Mg, P, N, S, Fe, Mn, Cu, Zn, we normalized the data to the control (expressed every value of every parameter as a percentage of its corresponding control; control = 100%). The dendrograms sort the parameters and the treatments (control, IBA (10^−11^ M), IBA (10^−7^ M), Cd, Cd + IBA, IBA (10^−9^ M)) according to their similarity. The heatmap was made by using function heatmap.2. From gplots package [60] in R software 4.0.2 (22 June 2020) [61]. The function heatmap.2 uses Euclidean distance measure to obtain distance matrix and complete linkage method for clustering.

Correlation matrix heatmap shows the values of Pearson correlation coefficient. The Pearson correlation coefficient is a measure of the strength of the linear relationship between two variables [62]. The correlation matrix was reordered according to the correlation coefficient, which helps to reveal hidden patterns in the matrix. The variables analyzed in the correlation matrix heatmap were elongation of PR, branching of PR, number of lateral roots (LR), fresh and dry weight (FW and DW), the activity of enzymes APX, CAT, SOD, the concentration of H_2_O_2_, the concentrations of nutrients Ca, K, Mg, P, N, S, Fe, Mn, Cu, Zn, the concentration of Cd. The correlation matrix heatmap was made using R software 4.0.2 (22 June 2020) (R Core Team 2020) and ggplot2 package [63].

### 4.7. Statistical Analyses

The data are displayed as mean values ± standard error (SE) and the experiments were repeated at least 3 times. Statistical analyses followed the analysis of variance (ANOVA) and Tukey test at *p* ˂ 0.05 with R software 3.6.3 (29 February 2020). However, for the parameter of the concentration of Cd, a Student’s *t*-test was used for the statistical analysis (*p* ˂ 0.05). The number of analyzed values depended on the parameters: 45 values for every treatment (analysis of the growth parameters), 15 for every treatment (analysis of the enzyme activity and the concentration of H_2_O_2_), and 9 for every treatment (analysis of the selected mineral nutrients).

## 5. Conclusions

**Figure 11:**
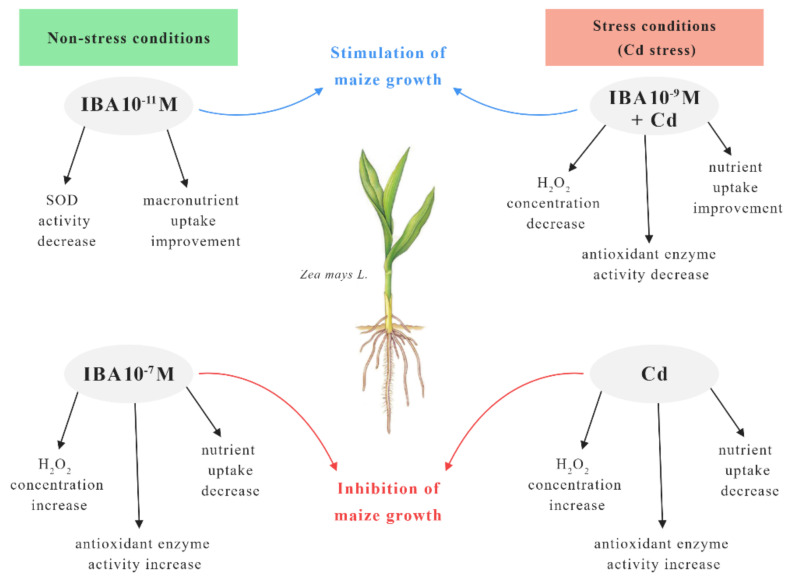
The scheme of the effects of IBA concentrations on maize plants in non-stress and stress (Cd stress) conditions.

Our results showed that exogenously applied IBA can influence the growth of the maize and alleviate Cd toxicity. The effect of IBA alone on plants depends on the concentration used, because it may act as an inhibitor or a stimulator of plant growth while influencing the oxidative status of the plant. For better understanding, we used a scheme to summarize the effects of IBA concentrations on maize plants grown in non-stress and stress conditions (Cd stress) (Figure 11).

In the non-stress conditions, the most effective concentration of IBA in the stimulation of growth was 10^−11^ M. This concentration improved the growth mainly by the increase in the nutrient uptake and decrease in the SOD activity when compared to the control. On the other hand, the higher concentration of IBA (10^−7^ M) increased the concentration of H_2_O_2_ and the activity of antioxidant enzymes which resulted in the inhibited growth. The presence of Cd in the substrate decreased the concentrations of nutrients in roots and increased the concentration of H_2_O_2_ which resulted in oxidative stress. Cadmium treatment also showed elevated activity of antioxidant enzymes, and the changed physiological status of the plant caused an inhibition of the root growth. However, in stress conditions (Cd stress) IBA in 10^−9^ M concentration best improved the toxic effects of Cd in plants. Application of IBA in 10^−9^ M decreased the concentration of H_2_O_2_ as well as the activity of antioxidant enzymes in plants and improved the uptake of essential nutrients. All mentioned changes resulted in improved growth of the plants when compared to Cd treatment.

## Data Availability

The data presented in this study are available on reasonable request from the corresponding author.
